# Recombinant expression and functional analysis of a *Chlamydomonas reinhardtii* bacterial-type phosphoenolpyruvate carboxylase gene fragment

**DOI:** 10.1007/s10529-013-1418-9

**Published:** 2013-12-29

**Authors:** Qi-Lin Tian, Ding-Ji Shi, Xiao-Hui Jia, Hua-Ling Mi, Xi-Wen Huang, Pei-Min He

**Affiliations:** 1 College of Fisheries & Life Sciences, Shanghai Ocean University, No. 999, Hucheng Ring Road, Shanghai, China; 2 Institute of Botany, Chinese Academy of Sciences, No. 20, Nanxin Village, Beijing, China; 3 National Key Laboratory of Plant Molecular Genetics, Institute of Plant Physiology and Ecology, Shanghai Institutes for Biological Sciences, Chinese Academy of Sciences, No. 300, Fenglin Road, Shanghai, China

**Keywords:** Algal lipids, *Chlamydomonas reinhardtii*, *Escherichia coli*, Gene expression, Lipid production, Phosphoenolpyruvate carboxylase, Reverse genetics

## Abstract

To investigate the function of a bacterial-type phosphoenolpyruvate carboxylase (PEPC2) derived from photosynthetically-grown *Chlamydomonas reinhardtii,* a fragment of the *pepc*2 gene was cloned and expressed in *Escherichia coli*. After optimal induction for 6 h, PEPC activity in the reverse mutant was lower than wild type (0.9 vs. 1.7 U/mg protein), and soluble protein was also lower than wild type (119 vs. 186 mg/g dry wt). In contrast, the total lipid content was increased from 56 (in wild type) to 71 mg/g dry wt, despite the growth rate being slightly diminished. The changes in PEPC activity, soluble protein and total lipid in the forward mutant were the opposite (2.4 U/mg, 230 mg/g, and 44 mg/g dry wt, respectively). Together, these data indicate that PEPC may function as a metabolic pivot in the regulation of protein and lipid accumulation in this alga.

## Introduction

When expression of phosphoenolpyruvate carboxylase (PEPC) in *Brassica napus* (Canola) was inhibited by its anti-sense RNA, the seed lipid content increased from 42 to 49 %, suggesting that PEPC may be involved in lipid accumulation (Chen et al. 1999). While it is yet to be confirmed whether the anti-sense RNA technique can be applied to prokaryotes, site-directed mutagenesis remains the method of choice for knockout of target genes in prokaryotic organisms (Terada et al. 1991). However, although site-directed mutagenesis has been instrumental for mechanistic studies, it often interferes with cell growth and is therefore not always suitable for industrial applications. In this study, we used a fragment of the *Chlamydomonas reinhardtii pepc*2 phosphoenolpyruvate carboxylase gene to down-regulate the expression of the *Escherichia coli* *pepc* gene using the reverse vectors method.

Phosphoenolpyruvate carboxylase is a key enzyme in plant metabolic pathways (O’Leary et al. 2011), catalyzing the irreversible carboxylation of phosphoenolpyruvate to oxaloacetic acid (OAA) in both the C4 and crassulacean acid carbon fixation, photosynthetic metabolic pathways. The enzyme is present not only in higher plants but also in prokaryotic bacteria, cyanobacteria, eukaryotic green algae and diatoms (O’Leary et al. 2011). Some eukaryotes contain plant- and bacterial-type PEPC enzymes (Sanchez and Cejudo 2003). The unicellular green alga, *C.* *reinhardtii,* grows photosynthetically and its genome sequence has been published (Merchant et al. 2007), and it is frequently used as a model organism for algal gene manipulation. Tarlan and Mammedov (2005) reported that *C. reinhardtii* has two PEPC genes; *pepc*1 encodes a plant-type enzyme whereas *pepc*2 encodes a bacterial-type enzyme. While *pepc*1 has been well studied, the function of *pepc*2 is not clear, and was investigated in this work.

The application of synthetic biology requires minimization of the genome and many of the genes within it. Since the *C. reinhardtii* *pepc*2 gene at 4,776 bp is relatively large (Tarlan and Mamedov 2005), it is not convenient to use the entire gene for transformation. Based on sequence analysis, a 639 bp fragment (about 1/7 of the full length gene), including only the first conserved sequence region and one of the two active sites, was cloned, heterologously expressed in *E. coli*, and characterized.

## Materials and methods

### Strains and plasmids

*Chlamydomonas reinhardtii* (FACHB-479) was from the Institute of Hydrobiology, Chinese Academy of Sciences (Wuhan, China), and grown at 25 °C in TAP medium under LL conditions (25 μmol photons m^−2^s^−1^). *E. coli* strain BL21 (DE3) pLysS was from TransGen Biotech (Beijing, China). Vector pET-28a was from Beijing Dingguo Changsheng Biochenology Co. Ltd (Beijing, China).

### *pepc*2 cloning and construction of expression vectors

**Fig. 1:**
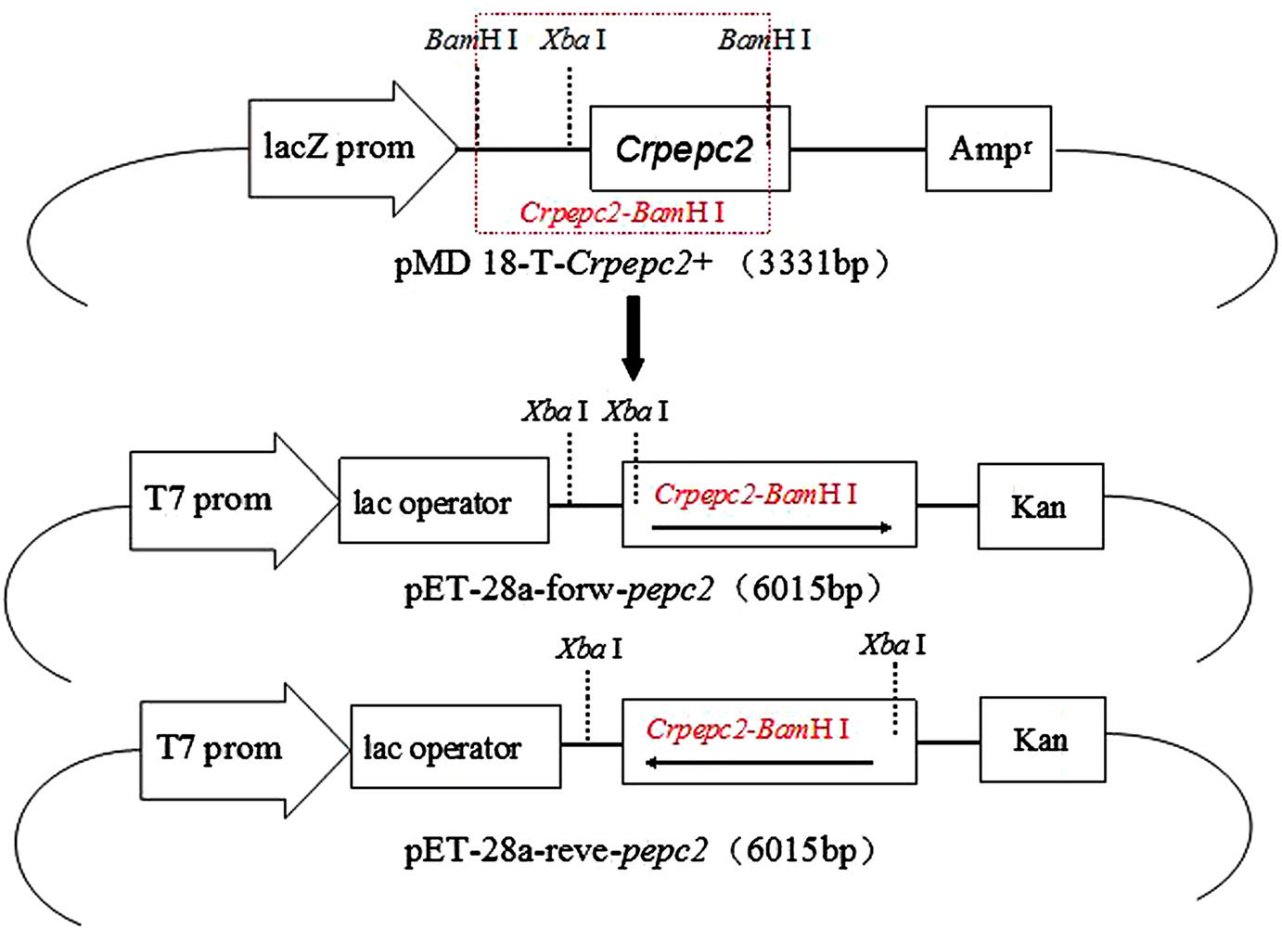
Construction of recombinant vectors pET-28a-forw-*pepc*2 and pET-28a-reve–*pepc*2. The *Chlamydomonas reinhardtii* *pepc*2 (*Crpepc*2) fragment was ligated into pMD18-T to construct the intermediate vector pMD18-T-*Crpepc*2. The forward vector pMD18-T-*Crpepc*2 was digested with *Bam*HI, and the fragment was inserted into plasmid pET-28a to generate pET-28a-*pepc*2. Digestion with *Xba*I distinguished between forward vector pET-28a-forw-*pepc*2 and reverse vector pET-28a-reve-*pepc*2

*Chlamydomonas reinhardtii* total RNA was extracted using Trizol and reverse transcribed using a reverse transcription kit. Primers P1 (CGATGCTCGGTAGCCTGCTTGACG; ATG underlined) and P2 (TAGGGATCCACAACGACTGCTCCACA; *Bam*HI underlined) were designed based on the *C. reinhardtii* *pepc*2 mRNA sequence (Genbank accession number AY517643). PCR was carried out as follows: denaturation at 94 °C for 5 min, followed by 32 cycles of 94 °C for 30 s, 60 °C for 30 s, 72 °C for 60 s, and a final extension of 72 °C for 5 min. The resultant *pepc*2 fragment (*Crpepc*2) was ligated into the pET-28a vector to make the recombinant forward construct pET-28a-forw-*pepc*2, and the reverse construct pET-28a-reve–*pepc*2 (Fig. 1).

### Transformation, induction, and growth

*Escherichia coli* BL21 (DE3) pLysS cells were transformed with either pET-28a (control mutant), pET-28a-forw-*pepc*2 (forward mutant) or pET-28a-reve-*pepc*2 (reverse mutant). Wild type and three mutant strains were inoculated into 5 ml LB medium and incubated at 37 °C overnight, respectively. Cultures were diluted 1:50 into fresh LB and grown at 25 °C for 4 h. When the OD_600_ reached 0.7, IPTG was added to 0.8 mM to induce *pepc*2 expression for 2, 4, 6, and 8 h, respectively. Growth rates were measured by recording the OD_600_ every 2 h. Cell cultures (10 ml) were collected, freeze-dried, weighed, and values were used to plot growth curves.

### PEPC activity, lipid and soluble protein content

Phosphoenolpyruvate carboxylase activity was measured at 25 °C by recording the decrease in absorbance at 340 nm using a PEPC kit [Comin Biotechnology (Suzhou) Co., Ltd, China]. PEPC activity was calculated using the following formula${{\left[ {\left( {\Delta {\text{A}} - \Delta {\text{A'}}_{\text{reference}} } \right) \times 4.94} \right]} \mathord{\left/ {\vphantom {{\left[ {\left( {\Delta {\text{A}} - \Delta {\text{A'}}_{\text{reference}} } \right) \times 4.94} \right]} {{\text{C}}_{\text{protein concentration}} }}} \right. \kern-0pt} {{\text{C}}_{\text{protein concentration}} }}$.

After collecting and freeze drying, total lipid was extracted using 2:1 methanol:chloroform (v/v). Total lipid was calculated by weighing as described by Bligh and Dyer (1959).

Soluble protein was isolated from the pelleted cells using lysate buffer (50 mM Tris, 1 mM EDTA, 0.1 mM NaCl, pH 8), and protein concentration was measured by the Bradford method. Total soluble protein was calculated using the following formula: ${{\left( {{\text{C}}_{\text{protein concentration}} \times {\text{V}}} \right)} \mathord{\left/ {\vphantom {{\left( {{\text{C}}_{\text{protein concentration}} \times {\text{V}}} \right)} {{\text{m}}_{\text{weight of dry cell}} }}} \right. \kern-0pt} {{\text{m}}_{\text{weight of dry cell}} }}$.

### RT-qPCR

After IPTG induction for 6 h, cells were harvested and total RNA was isolated and reverse transcribed as described above. Based on the DNA sequence of the MG1655 *pepc* gene in *E. coli* strain K-12 (Genbank accession number NC_000913.2), the forward primer P3 (AAAGCACGGCAGGATTAG) and the reverse primer P4 (TTGAGCAGGTTGCG-GTGT) were designed. Primers for the *gapdh* reference gene were provided by Bai et al. (2011). RT-qPCR was carried out using the SuperReal Premix Plus (SYBR Green) kit [Tiangen Biotech (Beijing) Co., Ltd, China] according to the manufacturer’s procedures.

## Results

### *pepc*2 cloning and construction of expression vectors

**Fig. 2:**
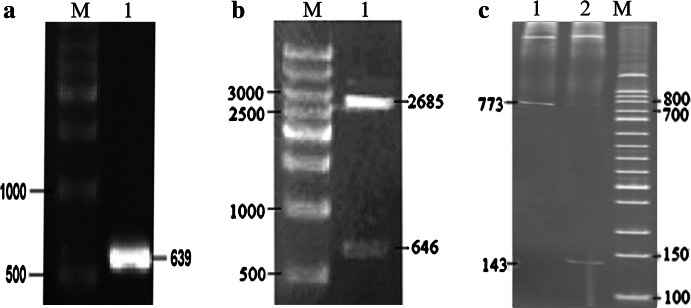
**a** Cloning of the *pepc*2 gene fragment.* M* 500 bp marker; *lane 1*, *pepc*2 gene fragment. **b** Identification of the forward vector pMD-18-T-*Crpepc*2 by *Bam*HI digestion.* M* 500 bp marker; *lane 1*, pMD18-T-*Crpepc*2+digested with *Bam*HI. **c** Identification of forward vector pET-28a-forw-*pepc*2 and reverse vector pET-28a-reve-*pepc*2 by *Xba*I digestion. *Lane 1*, pET-28a-reve-*pepc*2 digested with *Xba*I; *lane 2*, pET-28a-forw-*pepc*2 digested with *Xba*I;* M* 500 bp marker

A 639 bp fragment of *Crpepc*2 was amplified by PCR (Fig. 2a). This fragment, constituted a 1/7 of the entire *Crpepc*2 gene and included the ATG initiation codon, the first conserved sequence (VlTAHPTQalRR), and one of the catalytic domains (His^156^). This fragment was ligated to the plasmid pMD18-T and confirmed by DNA sequencing. The cloned fragment was 99 % identical to the GeneBank sequence, and the 1 % discrepancy was due to the *Bam*HI site incorporated in primer P2.

The intermediate vector pMD18-T-*Crpepc*2 included both the forward and reverse vectors, and digestion of the forward vector with *Bam*HI resulted in two fragments (2,685 and 646 bp) whereas digestion of the reverse vector with *Bam*HI resulted in a single 3,313 bp band (Fig. 2b). The 646 bp fragment (named *Crpepc*2-*Bam*HI) was inserted into pET-28a, and both the forward pET-28a-forw-*pepc*2 and the reverse pET-28a-reve-*pepc*2 expression vectors were obtained. Because *Crpepc*2-*Bam*HI contained an *Xba*I site in front of the gene fragment and the pET-28a vector also contains an *Xba*I site, digestion of the forward vector with *Xba*I resulted in a 143 bp fragment, whereas digestion of the reverse vector resulted in a 773 bp fragment (Fig. 2c).

### PEPC activity, total lipid and soluble protein, and cell growth

**Fig. 3:**
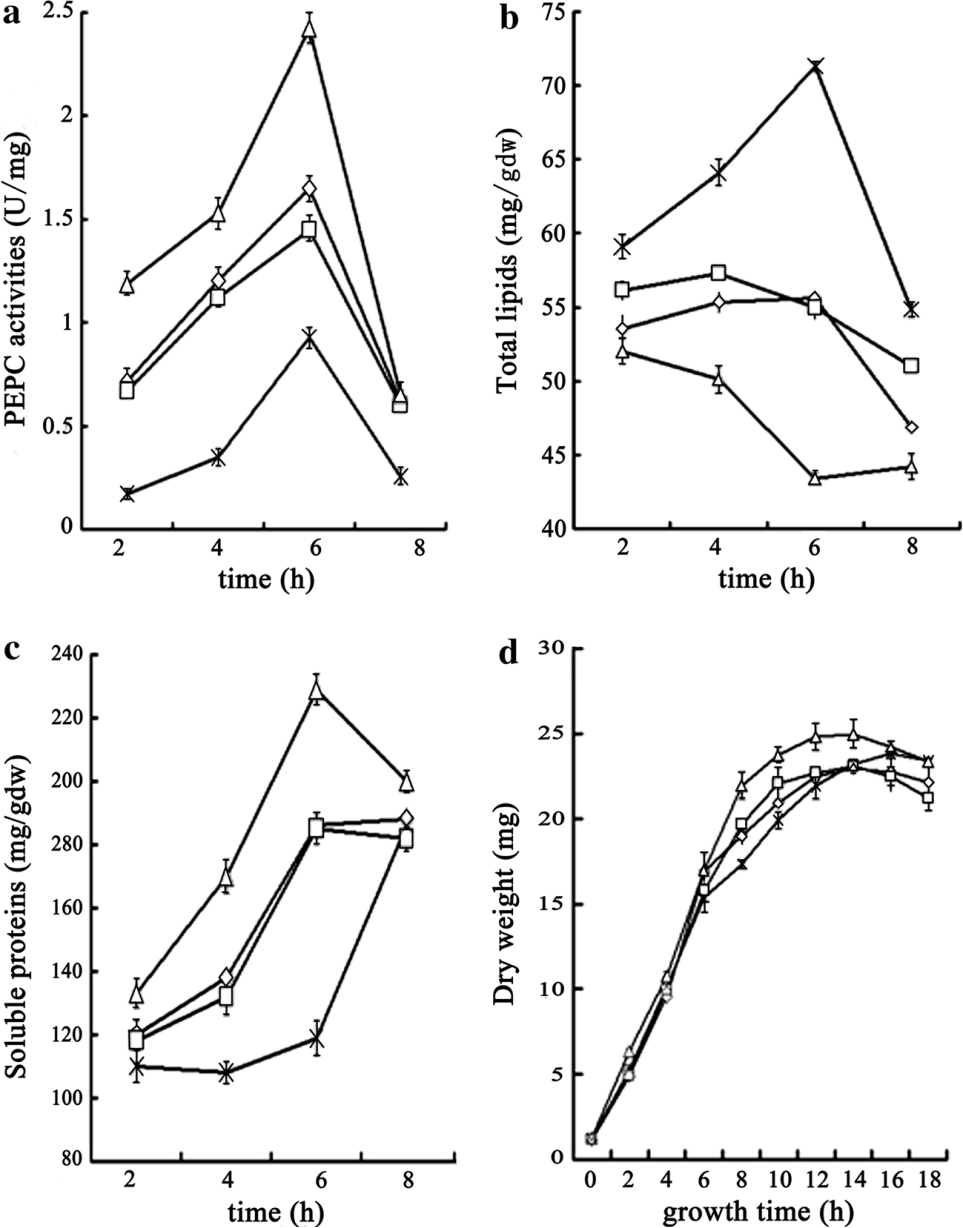
Characterization of wild type (*open* *diamond*), control (*open square*), forward (*open triangle*) and reverse (*times*) mutants. **a** PEPC activity, **b** total lipid content, **c** total soluble protein, **d** growth curve. Values presented are the mean±SD of three replicates

Phosphoenolpyruvate carboxylase activity, total lipid content, and total soluble protein of the four *E. coli* sub-strains (wild-type, pet28a, pET-28a-forw-*pepc*2, pET-28a-reve-*pepc*2) were measured following induction. The measurement results for the pet28a control strain were comparable to wild-type, as expected (Fig. 3). Interestingly, in the reverse mutant, PEPC activity was markedly decreased and only reached 0.2 U/mg protein after induction for 2 h, which was only 24 % that of wild type (*P* < 0.01). Total soluble protein was also decreased (119 mg/g dry wt after induction for 6 h), which was only 64 % of that of wild type (*P* < 0.01), although total lipid content was significantly increased, and reached a maximum of 71 mg/g dry wt after induction for 6 h, which was 128 % of that of wild type (*P* < 0.01). In contrast, both PEPC activity and total soluble protein were elevated in the forward mutant (2.4 U/mg and 230 mg/g dry wt, respectively, after induction for 6 h), which were 47 and 24 % higher than wild type, respectively (*P* < 0.01). Also in contrast to the reverse mutant, total lipid content was decreased (44 mg/g dry wt after induction for 6 h), which was 78 % that of wild type (*P* < 0.01). Importantly, neither forward nor reverse mutants significantly impacted the growth rate (Fig. 3). After 10 h of total growth (6 h after induction), the growth rate of the forward and the reverse mutants was 114 and 95 % that of wild type, respectively (*P* < 0.01).

### Relative expression of the *pepc* gene

The relative expression of *pepc* in the four sub-strains 6 h after induction was measured by RT-qPCR. Expression of *pepc* in wild type was taken as 100 %, and the expression of *pepc* in the forward and reverse mutants were 362 and 29 % of that of wild type, respectively (Table 1). The significantly increased and decreased *pepc* expression in the forward and reverse mutants, respectively, was statistically significant (*P* < 0.01).

**Table 1:** Relative expression levels of *pepc* in wild type, control, forward and reverse mutants

Strains	Ct^a^ (*gapdh* ^b^)	Ct^a^ (*pepc*)	Relative expression^c^ (%)
Wild type	27.03 ± 0.14	32.26 ± 0.18	100
Control mutant	27.30 ± 0.01	32.71 ± 0.37	88
Forward mutant	27.32 ± 0.02	30.70 ± 0.13	362
Reverse mutant	27.19 ± 0.04	34.23 ± 0.22	29

Samples were measured at 6 h after IPTG induction via RT-qPCR

Values presented are the mean of three replicates

^a^Ct was obtained from the amplification curves qPCR experiments

^b^Glyceraldehyde-3-phosphate dehydrogenase (*gapdh*) was used as a reference gene

^c^Relative expression was calculated as 2^−∆∆Ct^ ∆∆Ct = [Ct_mutant (*pepc*) − _Ct_mutant (*gapdh*)_] − [Ct_wild type (*pepc*) – _Ct_wild type (*gapdh*)_] and the  % expression of mutant strains is expressed relative to that of wild type, which was taken as 100 %

## Discussion

Our results raise three important points: (1) PEPC may play a key role as a metabolic pivot in controlling the directions of some metabolism pathways. For example, up-regulation of *pepc* expression may lead to increased amino acid biosynthesis and protein accumulation, whereas down-regulation appears to result in increased lipid accumulation. (2) Use of only a small portion (1/7) of the *C. reinhardtii* *pepc*2 gene was effective at down-regulating the expression of the *E. coli* *pepc* gene in the reverse mutant, as observed in previous RNAi experiments (Deng et al. 2011), despite a comparable growth rate to wild type cells. (3) Up- and down-regulation of *E. coli* was controlled by algal *pepc*2, suggesting a close structural homogeneity between the prokaryotic and eukaryotic enzymes. These results suggest the reverse vector method could prove to be an effective reverse genetic method for functional studies and also for potential industrial applications such as engineering organisms for biodiesel production.

Gene knockout and knockdown are powerful and widely used methods for studying gene function. Often, gene function can be determined by routine transformation and heterologous expression in model organisms such as *E. coli*. However, progress in reverse genetic studies has been much slower in prokaryotes than in eukaryotic organisms, which limits *E. coli* as a model organism for functional genomics experiments. About 15 years ago, we began investigating reverse genetics approaches in *E. coli* using the *glnA* glutamine synthetase gene (Qin et al. 1999). Although progress was made, our previous studies were restricted to prokaryotic genes (Hou et al. 2008; Jia et al. 2012). In this study, we have demonstrated that eukaryotic algal *pepc*2 is capable of downregulating the expression of *E. coli* *pepc* through reverse genetics.

The sequences of most eukaryotic genes are relatively long, and the capacity of prokaryotic expression vectors is often limited. Choosing the functionally important gene fragment is essential for successful application of the reverse vector method. The 639 bp fragment of *pepc*2 used in this study was chosen with reference to the molecular evolution study performed by Gehrig et al. (1998).

The maximal increase in total lipid content was 28 %, which was somewhat lower than that reported by Hou et al. (2008), who found that transformation of *E.coli* with *Anabaena* 7,120 *pepc* with a reverse expression vector increased total lipid content by 47 %. This difference may be associated with the degree of identity between the two gene sequences; *C. reinhardtii* *pepc*2 and *E. coli* *pepc* are 65 % identical, whereas *Anabaena* 7,120 and *E. coli* *pepc* gene are 72 % identical.

The catalytic activity of PEPC is not directly involved in fatty acid biosynthesis. Down-regulation of *pepc* decreases oxaloacetate (OAA), a precursor in amino acid biosynthesis, and increases pyruvate and acetyl-CoA, key precursors in fatty acid biosynthesis (Beck et al. 2012). Taken together, our results indicate that down-regulation of *pepc* expression may up-regulate pyruvate kinase and hence pyruvate levels, pyruvate dehydrogenases that catalyze the biosynthesis of acetyl-CoA, and acetyl-CoA carboxylase that directly synthesizes acetyl-CoA (Beck et al. 2012). PEPC may therefore function as a metabolic pivot that regulates multiple pathways simultaneously. A better understanding of the role of PEPC in microalgal metabolic pathways may prove useful for industrial applications such as biodiesel production.
